# Supplementary material for: Prevalence and molecular characterization of Entamoeba moshkovskii in diarrheal patients from Eastern India
Source: PLoS Negl Trop Dis. 2023 May 11;17(5):e0011287. doi: 10.1371/journal.pntd.0011287 (PMC10218735; doi:10.1371/journal.pntd.0011287)
Supplement: S1 Table — (DOCX) [file pntd.0011287.s001.docx]

**Supplementary Table 1:** List of clinical isolates of *E. moshkovskii* in diarrheal patients obtained from the hospital-based surveillance study.

| Sl No. | Name of organism | Id no | Target locus | GenBank Submission Id | Percentage of variation with respect to Prototype (AF149906.1) | SNPs Position | Nature of infection |
| --- | --- | --- | --- | --- | --- | --- | --- |
|  |  |  |  |  |  |  |  |
| 1 | *E. moshkovskii* | EM_IND/1 | 18S rRNA | ON965383 | 0% | NA | Sole D |
| 2 | *E. moshkovskii* | EM_IND/2 | 18S rRNA | ON965384 | 0% | NA | Sole D |
| 3 | *E. moshkovskii* | EM_IND/3 | 18S rRNA | ON965385 | 0.41% | 1345 T/G, 1361 A/G, 1377 T/G | Sole D |
| 4 | *E. moshkovskii* | EM_IND/4 | 18S rRNA | ON965386 | 0% | NA | Sole D |
| 5 | *E. moshkovskii* | EM_IND/5 | 18S rRNA | ON965387 | 0% | NA | Sole D |
| 6 | *E. moshkovskii* | EM_IND/6 | 18S rRNA | ON965388 | 0% | NA | Sole D |
| 7 | *E. moshkovskii* | EM_IND/7 | 18S rRNA | ON965389 | 0.14% | 988 G/C | Sole D |
| 8 | *E. moshkovskii* | EM_IND/8 | 18S rRNA | ON965390 | 0% | NA | Sole D |
| 9 | *E. moshkovskii* | EM_IND/9 | 18S rRNA | ON965391 | 0% | NA | Sole D |
| 10 | *E. moshkovskii* | EM_IND/10 | 18S rRNA | ON965391 | 0.14% | 722 T/C | Sole D |
| 11 | *E. moshkovskii* | EM_IND/11 | 18S rRNA | ON965392 | 0% | NA | Sole D |
| 12 | *E. moshkovskii* | EM_IND/12 | 18S rRNA | ON965393 | 0% | NA | Sole D |
| 13 | *E. moshkovskii* | EM_IND/13 | 18S rRNA | ON965394 | 0% | NA | Sole D |
| 14 | *E. moshkovskii* | EM_IND/14 | 18S rRNA | ON965395 | 0.41% | 1345 T/G, 1361 A/G, 1377 T/G | Sole D |
| 15 | *E. moshkovskii* | EM_IND/15 | 18S rRNA | ON965396 | 0.27% | 1345 T/G, 1361 A/G | Sole D |
| 16 | *E. moshkovskii* | EM_IND/16 | 18S rRNA | ON965397 | 0% | NA | Sole D |
| 17 | *E. moshkovskii* | EM_IND/17 | 18S rRNA | ON965398 | 0% | NA |  |
| 18 | *E. moshkovskii* | EM_IND/18 | 18S rRNA | ON965399 | 0.27% | 814 T/G,  826 T/A | Sole D |
| 19 | *E. moshkovskii* | EM_IND/19 | 18S rRNA | ON965401 | 0.41% | 1345 T/G, 1361 A/G, 1377 T/G | Sole D |
| 20 | *E. moshkovskii* | EM_IND/20 | 18S rRNA | ON965402 | 0% | NA | Sole D |
| 21 | *E. moshkovskii* | EM_IND/21 | 18S rRNA | ON965403 | 0.27% | 1345 T/G, 1361 A/G | Sole D |
| 22 | *E. moshkovskii* | EM_IND/22 | 18S rRNA | ON965404 | 0% | NA | Sole D |
| 23 | *E. moshkovskii* | EM_IND/23 | 18S rRNA | ON965405 | 0.14% | 788 T/C | Sole D |
| 24 | *E. moshkovskii* | EM_IND/24 | 18S rRNA | ON965406 | 0% | NA | Sole D |
| 25 | *E. moshkovskii* | EM_IND/25 | 18S rRNA | ON965407 | 0.41% | 1345 T/G, 1361 A/G, 1377 T/G | Sole D |
| 26 | *E. moshkovskii* | EM_IND/26 | 18S rRNA | ON965408 | 0% | NA | Sole D |
| 27 | *E. moshkovskii* | EM_IND/27 | 18S rRNA | ON965409 | 0% | NA | Sole D |
| 28 | *E. moshkovskii* | EM_IND/28 | 18S rRNA | ON965410 | 0% | NA | Sole D |
| 29 | *E. moshkovskii* | EM_IND/29 | 18S rRNA | ON965411 | 0.41% | 1345 T/G, 1361 A/G, 1377 T/G | Sole D |
| 30 | *E. moshkovskii* | EM_IND/52 | 18S rRNA | ON965412 | 0.14% | 1145 G/A | SOLE D |
|  | | | | | | | |
| 31 | *E. moshkovskii* | EM_IND/31 | 18S rRNA | ON965413 | 0% | NA | IB/V |
| 32 | *E. moshkovskii* | EM_IND/32 | 18S rRNA | ON965414 | 0.27% | 814 T/G  826 T/A | IB/V |
| 33 | *E. moshkovskii* | EM_IND/33 | 18S rRNA | ON965415 | 0.27% | 814 T/G  826 T/A | IB/V |
| 34 | *E. moshkovskii* | EM_IND/34 | 18S rRNA | ON965416 | 0% | NA | IB/V |
| 35 | *E. moshkovskii* | EM_IND/35 | 18S rRNA | ON965417 | 0% | NA | IB/V |
| 36 | *E. moshkovskii* | EM_IND/36 | 18S rRNA | ON965418 | 0% | NA | IB/V |
| 37 | *E. moshkovskii* | EM_IND/37 | 18S rRNA | ON965419 | 0.27% | 769 A delete  795-796 T insert | IB/V |
| 38 | *E. moshkovskii* | EM_IND/38 | 18S rRNA | ON965420 | 0.27% | 814 T/G  826 T/A | IB/V |
| 39 | *E. moshkovskii* | EM_IND/39 | 18S rRNA | ON965421 | 0.27% | 814 T/G  826 T/A | IB/V |
| 40 | *E. moshkovskii* | EM_IND/40 | 18S rRNA | ON965422 | 0.14% | 1437 G/A | IB/V |
| 41 | *E. moshkovskii* | EM_IND/41 | 18S rRNA | ON965423 | 0% | NA | IB/V |
| 42 | *E. moshkovskii* | EM_IND/42 | 18S rRNA | ON965424 | 0% | NA | IB/V |
| 43 | *E. moshkovskii* | EM_IND/43 | 18S rRNA | ON965425 | 0% | NA | IB/V |
| 44 | *E. moshkovskii* | EM_IND/44 | 18S rRNA | ON965426 | 0.27% | 814 T/G  826 T/A | IB/V |
| 45 | *E. moshkovskii* | EM_IND/45 | 18S rRNA | ON965426 | 0% | NA | IB/V |
| 46 | *E. moshkovskii* | EM_IND/46 | 18S rRNA | ON965427 | 0.27% | 769 A delete,  795-796 T insert | IB/V |
| 47 | *E. moshkovskii* | EM_IND/47 | 18S rRNA | ON965428 | 0% | NA | IB/V |
| 48 | *E. moshkovskii* | EM_IND/48 | 18S rRNA | ON965429 | 0.27% | 814 T/G  826 T/A | IB/V |
| 49 | *E. moshkovskii* | EM_IND/49 | 18S rRNA | ON965430 | 0% | NA | IB/V |
| 50 | *E. moshkovskii* | EM_IND/50 | 18S rRNA | ON965431 | 0.27% | 814 T/G  826 T/A | IB/V |
| 51 | *E. moshkovskii* | EM_IND/51 | 18S rRNA | ON965432 |  | NA | IB/V |
|  | | | | | | | |
| 52 | *E. moshkovskii* | EM_IND/52 | 18S rRNA | ON965433 | 0.14% | 1145 G/A | ISTH |
| 53 | *E. moshkovskii* | EM_IND/53 | 18S rRNA | ON965434 | 0% | NA | ISTH |
| 54 | *E. moshkovskii* | EM_IND/54 | 18S rRNA | ON965435 | 0% | NA | ISTH |
| 55 | *E. moshkovskii* | EM_IND/55 | 18S rRNA | ON965436 | 0% | NA | ISTH |
| 56 | *E. moshkovskii* | EM_IND/56 | 18S rRNA | ON965437 | 0% | NA | ISTH |
|  | | | | | | | |
| 57 | *E. moshkovskii* | EM_IND/57 | 18S rRNA | ON965438 | 0.14% | 722 T/C | IEH |
| 58 | *E. moshkovskii* | EM_IND/58 | 18S rRNA | ON965439 | 0.14% | 722 T/C | IEH |
| 59 | *E. moshkovskii* | EM_IND/59 | 18S rRNA | ON965440 | 0.14% | 722 T/C | IEH |
| 60 | *E. moshkovskii* | EM_IND/60 | 18S rRNA | ON965441 | 0.27% | 814 T/G  826 T/A | IEH |
| 61 | *E. moshkovskii* | EM_IND/61 | 18S rRNA | ON965442 | 0.27% | 814 T/G  826 T/A | IEH |
| 62 | *E. moshkovskii* | EM_IND/62 | 18S rRNA | ON965443 | 0.14% | 722 T/C | IEH |
|  | | | | | | | |
| 63 | *E. moshkovskii* | EM_IND/63 | 18S rRNA | ON965445 | 0% | NA | IOEP |
| 64 | *E. moshkovskii* | EM_IND/ 64 | 18S rRNA | ON965446 | 0.14% | 1437 G/A | IOEP |
| 65 | *E. moshkovskii* | EM_IND/65 | 18S rRNA | ON965447 | 0% | NA | IOEP |
| 66 | *E. moshkovskii* | EM_IND/66 | 18S rRNA | ON965448 | 0% | NA | IOEP |
| 67 | *E. moshkovskii* | EM_IND/67 | 18S rRNA | ON965449 | 0.14% | 988 G/C | IOEP |
| 68 | *E. moshkovskii* | EM_IND/68 | 18S rRNA | ON965450 | 0% | NA | IOEP |

Sole D : diarrheal patients solely infected with E. moshkovskii, IEH: *E moshkovskii* positive samples co-infected with *Entamoeba histolytica*, IOEP: *E moshkovskii* positive samples co-infected with other Enteric Parasites- *G. lamblia*, *Cryptosporidium* spp, ISTH *: E moshkovskii* positive samples co-infected with soil transmitted helminths, IB/V: *E moshkovskii* positive samples co-infected with other diarrhea causing bacteria-*E. coli*, *Shigella* spp & *V. cholera* or virus-Rotavirus.
