# Supplementary material for: Prevalence and molecular characterization of Entamoeba moshkovskii in diarrheal patients from Eastern India
Source: PLoS Negl Trop Dis. 2023 May 11;17(5):e0011287. doi: 10.1371/journal.pntd.0011287 (PMC10218735; doi:10.1371/journal.pntd.0011287)
Supplement: S2 Table — (DOCX) [file pntd.0011287.s002.docx]

**Supplementary Table 2:** Substitution matrix based on 18S rRNA sequences of the populations of *E. moshkovskii* collected in and around Kolkata.

|  | **A** | **T/U** | **C** | **G** |
| --- | --- | --- | --- | --- |
| **A** | - | *5.33* | *4.10* | **7.63** |
| **T/U** | *7.02* | - | **16.83** | *5.50* |
| **C** | *7.02* | **21.89** | - | *5.50* |
| **G** | **9.74** | *5.33* | *4.10* | - |

Each entry is the probability of substitution (*r*) from one base (row) to another base (column). Substitution pattern and rates were estimated under the Tamura-Nei (1993) model (+I). The rate variation model allowed for some sites to be evolutionarily invariable ([+*I*], 45% sites). Rates of different transitional substitutions are shown in **bold** and those of transversionsal substitutions are shown in *italics*. Relative values of instantaneous *r* should be considered when evaluating them. For estimating ML values, a tree topology was automatically computed. This evolutionary analysis was conducted in MEGA X.
