# Supplementary material for: Prevalence and molecular characterization of Entamoeba moshkovskii in diarrheal patients from Eastern India
Source: PLoS Negl Trop Dis. 2023 May 11;17(5):e0011287. doi: 10.1371/journal.pntd.0011287 (PMC10218735; doi:10.1371/journal.pntd.0011287)
Supplement: Attachment — Submitted filename: reviewer response plos ntd em779.pdf [file pntd.0011287.s003.pdf]

To,
The Editor-in-Chief,
PLOS Neglected Tropical Diseases

Dear Editor,

We want to express our gratitude for the time and effort you put into reviewing our paper and providing valuable comments. Your feedback helped us to improve the manuscript significantly, and we are grateful for your insightful and constructive comments. We carefully considered each of your comments and made revisions accordingly to meet your high standards. If you have any further suggestions, we welcome them. Following are our answers to your comments.

Thank you for your time and support.

Best regards,

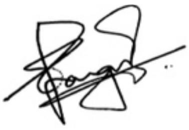

Dr. Sandipan Ganguly
Scientist F
ICMR-NICED, Kolkata
India

Comment-1:
* Despite the authors claiming ethical approval, I missed a protocol number that proves this information.

Our answer:
Thank you so much for bringing this point to our attention. We have mentioned the protocol number in the Ethical statement section.

Comment-2:
* In sentence (line 127): “Optimum extension temperature at 72°C gave highest intensity of amplicons than any other temperature.” The authors could clarify why they tested different extension temperatures in PCR. Does the Taq used allow other temperatures? If not, I suggest removing the sentence. Also, add Taq manufacturer (lines 128-130).

Our answer:
Thank you for pointing this out. We have removed the sentence as suggested. We have added the Taq manufacturer.

Comment-3:
* The authors propose a new tool for detecting E. moshkovskii based on nested PCR. Considering that the authors cultivate E. moshkovskii, why did they not determine the detection limit of the test?

Our answer:
Thank you for bringing this to our attention. The sensitivity of the PCR system was evaluated during the standardization process, and the results of this testing are now included in the updated version of the manuscript.

Comment-4:
* Did the authors test the new methodology for negative samples in microscopy (considering the higher sensitivity of nested PCR)?

Our answer:
Thank you for the insightful inquiry. We also carried out the PCR assay on the microscopy-negative samples. Kindly refer to the section on the **Prevalence of *Entamoeba moshkovskii*** in the results.

Comment-5:
* What criteria was used to choose the Tamura Nei model?
Ans: Thank you very much for your query. We used this model because it is recognized as a robust method for constructing phylogenetic trees. Moreover, this model has been shown to produce more accurate results compared to other models in reconstructing evolutionary relationships among species, particularly for non-coding regions of DNA where other models may perform poorly.

Comment-6:
* It is unclear how GPS data was acquired in the study. Please clarify.

Our answer:
Thank you for pointing this out. The geographical locations of the patients were obtained from the hospital case record file (CRF). The information has been included in the GIS analysis section.

Comment-7:
* Information that should be in the material and methods section (only) is in the results section (lines 240-241, 305-306). Please correct.

Our answer:
Thank you for the suggestion. The information has been integrated into the results section.

Comment-8:
* Please standardize: 18SrRNA or SSUrRNA

Our answer:
Thank you for the comment. All the SSUrRNA have been updated to 18S rRNA throughout the manuscript.

Comment-9:
* In Figure 9, the authors present a phylogenetic tree where the E. moshkovskii isolates grouped together in an (almost) monophyletic way, with a few almost imperceptible differences using the proposed methodology. However, they argue as if there were four different groups within the species. Furthermore, they state that two E. moshkovskii sequences “is classified as an out-group in a speciation process and showed one insertion (nucleotide A) and one deletion (Nucleotide T) at the studied locus” (line 318-319). I would like the authors to clarify the outgroup concept. Analyzes need to be interpreted appropriately.

Our answer:
We have striven to interpret the phylogenetic tree precisely. A revised clarification has been incorporated in the new manuscript. We welcome any additional feedback or suggestions for improvement.

Comment-10:
* I suggest performing a haplotype network analysis to clarify intraspecific differences.

Our answer:
Thank you for the suggestion. We have performed a haplotype network analysis and incorporated it in the new manuscript.

Comment-11:
* A point of concern is the correlation of SNPs vs. clinical features, especially when only one locus is analyzed. I would like the authors to point out other studies that have found this correlation using 18S rRNA locus.

Our answer:
Your comment is greatly appreciated. We could not find any previous articles that establish a correlation between 18S rRNA SNPs and clinical features. Our article may be the first attempt to investigate this relationship. Nevertheless, we have revised the discussion section to present the correlation in a different manner.

Comment-12:
Conclusions:
* Authors may "suggest" that E.moshkovskii causes diarrhea (lines 511-512).

Our answer:
Thank you for pointing this out. We have changed the lines accordingly.

Comment-13:
Editorial and Data Presentation Modifications:
Abstract section:
* Please, enter the total sample number.

Our answer:
Thank you for the suggestion. The total sample number has been included in the abstract section.

Comment-14:
* Replace: GenBank accession number “KP72260.51” by “KP722605.1”
Our answer:
Thank you for pointing this out. We have changed the GenBank accession number.

Comment-15:
Author summary section:
* Authors must reformulate the “Author summary” based on the PNTD guidelines (https://journals.plos.org/plosntds/s/submission-guidelines): “Distinct from the scientific abstract, the Author Summary should highlight where the work fits in a broader context of life science knowledge and why these findings are important to an audience that includes both scientists and non-scientists. Ideally aimed to a level of understanding of an undergraduate student, the significance of the work should be presented simply, objectively, and without exaggeration.”

Our answer:
Your suggestion is greatly appreciated. We have reformulated the “Author summary” in the current version based on the PNTD guidelines. The authors are open to receiving additional constructive feedback if any.

Comment-16:
Introduction section:
* I found the intro section to be quite large. But it's up to the authors.

Our answer:
Thank you for the comment. We made some grammatical changes. We have not made any changes to the length of the introduction as we want to keep it as it was originally.

Comment-17:
Material and Methods section: In general, the Material and Methods section should be carefully reviewed and written with greater scientific rigor.

Our answer:
We greatly appreciate the constructive comment. The material and method section underwent revision and all the modifications are highlighted.

Comments-18:
* Line 94: to correct “Enatomeba”.
*Line 105: add the species E. nuttalii and its GenBank accession number.
* Line 108: add E. coli GenBank accession number.
* Line 113: replace “18srRNA” by 18SrRNA”.
* Several times: replace “Mgcl2” by “MgCl2”
* Line 174 (suggestion): replace “using the BLAST program run on the NCBI website” by “using the BLAST tool (NCBI - https://blast.ncbi.nlm.nih.gov/Blast.cgi)”

Our answer:
Your suggestions are acknowledged and we have made the necessary changes.

Comment-19:
* The authors should add information on a possible second species, in lines 220-225: “These two strains were cultured at 25°C and 37°C, respectively. Trophozoites under log phase of growth were used in the experiments as positive control for molecular identification of both species.”

Our answer:
Thank you for bringing this to our attention. For this study, we maintained a single strain of *E. moshkovskii* and the culture details have been included in the latest version. The *E. moshkovskii* culture was maintained at 25°C in our laboratory. Although we also maintain the *E. histolytica* HM1:IMSS strain at 37°C, it is not relevant for this study. The revised manuscript now includes details of the *E. moshkovskii* culture.

Comments-20:
* Lines 174-175: add GenBank accession numbers obtained.
* Lines 179-180: Is it necessary to “ML” multiple times? in “Substitution matrix (ML) and transition/transversion (ML) bias were estimated using the same software. In substitution matrix (ML) each entry…”. What does ML mean?
* Line 182: [Reference]?
* Line 285: replace monsoon by fall.

Our answer:
We are grateful for your carefully considered and supportive remarks. The issues abovementioned have now been addressed in the revised manuscript.

Comment-21:
Summary and General Comments:
The manuscript “Prevalence and molecular characterization of Entamoeba moshkovskii in diarrheal patients from Eastern India” reports the finding of this species in human samples, an intriguing and understudied protozoan. The study demonstrates clear epidemiological importance and highlights the pathogenic potential of Entamoeba moshkovskii. The study brings new information and presents us with an expressive number of samples.
The authors identified the species E. moskhovskii through 18SrRNA nested PCR+sequencing and made a correlation between the polymorphisms found and the clinic. However, I have some reservations regarding correlations of single nucleotide polymorphisms being responsible for clinical features in eukaryote infections, especially when only one locus is analyzed.
Finally, a language review should be performed.

Our answer:
Thank you for your feedback; it is much appreciated. We have revised the discussion section regarding correlations of single nucleotide polymorphisms being responsible for clinical features (lines 568-574).

Reviewer's Responses to Questions

Comments-22:

Key Review Criteria Required for Acceptance?</br></br>
As you describe the new analyses required for acceptance, please consider the following:</br></br>

Methods</br></br>
-Are the objectives of the study clearly articulated with a clear testable hypothesis stated?</br>
-Is the study design appropriate to address the stated objectives?</br>
-Is the population clearly described and appropriate for the hypothesis being tested?</br>
-Is the sample size sufficient to ensure adequate power to address the hypothesis being tested?</br>
-Were correct statistical analysis used to support conclusions?</br>
-Are there concerns about ethical or regulatory requirements being met?</br></br>

Reviewer #1: Yes, the author did something, and described all contents in detail. And they did incomplete and be necessary to summarize really useful results from so many analyses.

Reviewer #2: The authors applies correct methodology, and the study design and materials are clearly laid out

----

Our answer:
We sincerely appreciate your positive feedback.

Comments-23:

Results</br></br>
-Does the analysis presented match the analysis plan?</br>
-Are the results clearly and completely presented?</br>
-Are the figures (Tables, Images) of sufficient quality for clarity?</br></br>

Reviewer #1: The manuscript contains a large number of figures and tables. Some of them contain little information,   for example Fig 2, Fig 3 and Fig 4 could be deleted. Fig 5, Fig 6 and Fig 7 could be composed to one figure. Fig 10 to 13 could be composed to one figure. Data of table 1, table 2, Table 6 and Table 7 are duplicated with figures. Table 3 and table 5 could submit as a supplementary file. Some figures were of poor quality, such as Fig 5, Fig 6, Fig 10 to 13. The author should use professional software to reproduce.

Reviewer #2: the results are clearly and completely presented. However, figure 9 is with a low resolution

----

Our answer:
We greatly appreciate your constructive comments.
We have deleted Fig 2, Fig 3 and Fig 4…. Fig 5, Fig 6 and Fig 7 have been composed into one figure as suggested. Tables 1, 2, and 7 have been removed due to duplication with the accompanying figures. We also removed figures 11-13 but kept Table 6. We prefer table 6 instead of its corresponding graphical presentation (Figure 10-12). We appreciate any additional constructive feedback regarding the tables and figures presented. We have made efforts to improve the quality of the images that were previously of poor quality.
We have increased the resolution (>300 dpi) of Figure 9 to provide a clearer visual representation.

Comments-24:
Conclusions</br></br>
-Are the conclusions supported by the data presented?</br>
-Are the limitations of analysis clearly described?</br>
-Do the authors discuss how these data can be helpful to advance our understanding of the topic under study?</br>
-Is public health relevance addressed?</br></br>

Reviewer #1: Yes, the author did something, it was necessary to summarize really key conclusions from so many analyses.

Reviewer #2: The authors wrote a conclusion is too long and sounding repeatitive.

The conclusion section is where authors a brief summary of the paper’s main points, but don’t simply repeat things that were in your paper. A conclusion is expressed in a few precisely worded sentences, usually one paragraph or 200 to 300 words and interpret your findings at a higher level of abstraction.

Our answer:
Thank you so much for the constructive comments.
We have summarized the conclusion section to present a concise summary of our findings.

Comments-25:

Editorial and Data Presentation Modifications?</br>
Use this section for editorial suggestions as well as relatively minor modifications of existing data that would enhance clarity. If the only modifications needed are minor and/or editorial, you may wish to recommend “Minor Revision” or “Accept”.

Reviewer #1: I will suggest performing major Revision

Reviewer #2: The manuscript has important at local scale, and should be of great interest to the readers from Kolkata and adjacent areas, Eastern India.
Findings are well presented and the manuscript present in an intelligible fashion but it is not written in standard English.
I recommend that authors send their manuscript for professional copy editing and rewrite a conclusion
The paper is recommended for Minor Revision

Our answer:

Thank you very much for your insightful comments. The manuscript was thoroughly revised to correct grammatical errors. We revised the conclusion section.

Comments-25:

Summary and General Comments</br></br>
Use this section to provide overall comments, discuss strengths/weaknesses of the study, novelty, significance, general execution and scholarship. You may also include additional comments for the author, including concerns about dual publication, research ethics, or publication ethics. If requesting major revision, please articulate the new experiments that are needed.

Reviewer #1: The manuscript reported high prevalence rate of sole infection with E. moshkovskii in diarrhoeal patients in two local hospitals in India. The investigation involved a large number of clinical samples and demonstrated that E. moshkovskii is one of the causative agents for acute diarrhea in human, and indicated E. moshkovskii had pathogenic potential. It is an interesting and meaningful molecular epidemiological study, but the manuscript needed to be major revised.

1. The author described all contents in detail, but the main results and conclusions of the article were not highlighted. Manuscripts can be greatly simplified.
2. The manuscript contains a large number of figures and tables. Some of them contain little information, for example, Fig 2, Fig 3 and Fig 4 could be deleted. Fig 5, Fig 6 and Fig 7 could be composed to one figure. Fig 10 to 13 could be composed to one figure. Data of table 1, table 2, Table 6 and Table 7 are duplicated with figures. Table 3 and table 5 could submit as a supplementary file.
3. Additionally, some figures were of poor quality, such as Fig 5, Fig 6, Fig 10 to 13. The author should use professional software to reproduce.
4. The author used many tools to analyze polymorphism of E. moshkovskii18S gene. The results seemed similar. In addition, figure 9 suggested high similarity in E. moshkovskii18S gene and the 18S gene was a conserved gene. It was necessary to summarize really useful results from so many analyses.

Reviewer #2: (No Response)

Our answer:

We sincerely appreciate your positive feedback. We have some changes in the results and conclusion sections as suggested by the reviewers and have tried to simplify the manuscript. We welcome any further constructive feedback.

We have deleted Fig 2, Fig 3 and Fig 4 as suggested. Fig 5, Fig 6 and Fig 7 have been composed into one figure as suggested. Tables 1, 2, and 7 have been removed due to duplication with the accompanying figures. We also removed figures 11-13 but kept Table 6. We prefer table 6 instead of its corresponding graphical presentation (Figure 10-12). Table 3 and Table 5 have been submitted as supplementary files.

We have tried to improve the quality of the images (Fig 5 & 6) that were previously of poor quality. Fig 10 has been deleted and table 2 has been added in its place.
Thank you for your insightful comments and for pointing out the shortcomings in the manuscript, we have tried our best to address each of them individually. If there is anything else you would like to ask or add, we would be more than happy to hear from you.

Comments-26:

PLOS authors have the option to publish the peer review history of their article (what does this mean?). If published, this will include your full peer review and any attached files.

If you choose “no”, your identity will remain anonymous but your review may still be made public.

Do you want your identity to be public for this peer review? For information about this choice, including consent withdrawal, please see our Privacy Policy.

Reviewer #1: No

Reviewer #2: No

Our answer:
We greatly appreciate the feedback you've provided.
