# Supplementary material for: Prevalence and molecular characterization of Entamoeba moshkovskii in diarrheal patients from Eastern India
Source: PLoS Negl Trop Dis. 2023 May 11;17(5):e0011287. doi: 10.1371/journal.pntd.0011287 (PMC10218735; doi:10.1371/journal.pntd.0011287)
Supplement: Attachment — Submitted filename: pdf PNTD response after acceptance.pdf [file pntd.0011287.s005.pdf]

To,
The Editor-in-Chief,
PLOS Neglected Tropical Diseases

Dear Editor,

I would like to express our gratitude for your efforts in managing the article and accepting our manuscript for publication. We would also like to extend our appreciation to the reviewers for their invaluable inputs, which enabled us to enhance the quality of the manuscript. We have revised the manuscript based on the suggestions provided by both the editor and the reviewers to improve its readability. If you have any additional suggestions, please do not hesitate to let us know.

Thank you for your time and continued support.

Best regards,

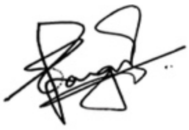

Dr. Sandipan Ganguly
Scientist F
ICMR-NICED, Kolkata
India

Comment-1:

The authors must correct all types grammatical error in manuscript. For instance, in line 563, replace "dirrhoeal" by diarrhoeal

Our answer:
Thank you so much for bringing this point to our attention. We have replaced "dirrhoeal" with diarrhoeal. We made some grammatical changes also.

Comment-2:

As mentioned by authors, in line 362 , the authors argued that " Interestingly, 100% (n/N=17/17, 95% CI 0.784-363 1.00) of trophozoites were found to be non-hematophagous, indicating that *E. moshkovskii* may cause a non invasive form of intestinal amoebic infection"

This information is redundant because of globally it is observed that 90% of E. histolytica infections are asymptomatic and only in case of invasive amebiasis is observed hematophagous trophozoites on stool microscopy.

Our answer:
Thank you for the suggestion. We have removed the sentence.

Comment-3:
In line 319 , item Data collection of other Enteropathogen infections:
The authors mention: " Entamoeba histolytica, Cryptosporidium, and Giardia were identified by PCR method as described elsewhere. Helminth parasites were detected by light microscopy after wet mount. Other enteric pathogens like Vibrio cholera O1/O139, Salmonella spp., Campylobacter jejuni, Rotavirus, astrovirus and adenovirus co-infection information with entamoeba were obtained from the institutional database of ICMR-National Institute of Cholera and Enteric Diseases, Kolkata, India. Detailed information is available elsewhere [41]".

This is reference is about a hospital-based laboratory surveillance study that it was conducted among the patients admitted between November 2007 and October 2008 to the Infectious Diseases (ID) Hospital (Population = 1103) with diarrhoeal complaints.

Could the author explain about it. Where is detailed information about of present paper data (hospital-based systemic surveillance data conducted from March 2017 to February 2020)?

In line 374, diarrheagenic organisms were screened in the *E. moshkovskii-positive* diarrheal cases. There are no mention about methodology for virus and bacteria detections. What were methods employed?

Our answer:
Thank you so much for bringing this point to our attention. The reference number 41 has been incorrectly placed and has now been corrected to its proper location on line 321. The identification of *Entamoeba histolytica, Cryptosporidium* spp, and *Giardia* was carried out according to the protocol outlined in the reference article (reference no. 41).
We retrieved information on the virus and bacterial infections associated with *E. moshkovskii*-positive samples from our institutional database. The Division of Microbiology and the Division of Virology at ICMR-NICED conducted the detection of the virus and bacteria and recorded the data in the institutional database. We obtained the data from there.

Comment-3:

In line 392 item GIS Mapping: The authors mentioned

"GIS mapping of the patients admitted to the I.D. hospital and B C Roy Children Hospital pointed out that the Beliaghata, Entally, Kashipur-Belgachia, Maniktala, Gopalpur-Rajarhat, Kolkata-Port, Jorasanko, Baruipurpurba, Maheshtala, Metiabruz, Bhangar areas of Kolkata and the adjacent regions were very highly infected (>3.5%) with *Entamoeba moshkovskii*. Ballygunge, Chowragee, Dumdum Uttar, Dumdum, Bidhannagar, Bhabanipur and Kashba had high prevalence rate (3%-3.5%). Behala Paschim, Behala Purba, Jadavpur were moderately (2.5%-2.99%) infected, while the lowest prevalence (< 2.49%) was reported in Baranagar, Rajarhat New Town and Shyampukur areas [Figure 3]. Those areas that constituted less than 1% of the total sample size have been excluded from the mapping".

I suggest put prevalence rate in figure GIS Mapping and the authors must reduce this paragraph in order to avoid replicate information. Images help readers visualize the information.

<u>Our answer:</u>

We recently discussed with Dr. Orsolya Voros, Journal Assistant, Editorial Office Ltd., PLOS Neglected Tropical Diseases, regarding the use of the basemap shapefile in our manuscript. Dr. Voros informed us that the shapefile is licensed under the Open Data Commons Open Database License (ODbL), which may prohibit us from publishing the map in PLOS NTD. Based on his feedback, we have decided to remove the map from the manuscript.

In light of this decision, we made some modifications to the methodology and results sections that were associated with the GIS mapping, to reflect the removal of the map. We are open to further suggestions and feedback from the editorial team.

As the figure is removed, we are unable to put the prevalence rate in figure GIS Mapping. Nonetheless, we would appreciate any additional feedback you may have on this alteration.

Comment-4:

*In line 408, the authors mentioned "*the rest of the 32 sequences represented genetic variants of *E. moshkovskii* not described earlier"

How many nucleotide sequences (genetic variants of E. moshkovskii) are currently avaliable by GenBank?

intraspecific polymorphism exist but this information should be analysed based on robust data.

Our answer:
Thank you for the insightful inquiry. We were able to obtain and analyze 10 haplotypes in this study region, with 9 of them containing 32 sequence variants and the remaining haplotype having 100% identity with the Laredo strain. Upon conducting a survey, we found a considerable number of sequence variants in the NCBI GenBank, making it difficult to estimate their exact number. We look forward to any additional feedback.

Comments-5:
In line 587 the authors mentioned that "SNPs in the 18S rRNA gene could have a significant impact on the biology of the amoeba"
Could the authors explain about it ? what is significant consequence ?

Could the authors place this sentence within the context of previous studies

In discussion section, the authors should compare and contrast to previous studies. And also, although interpretation is the primary goal of the Discussion section, authors must be careful not to overinterpret their data, or stray too far from scientific evidence.

The author should providing proper context for your research and avoiding introducing new information

For instances, in line 592 "*E. moshkovskii* infection may alter susceptibility to infection with other enteric pathogens and modulate the effects of co-infecting gut pathogens".
In line 593 "*E. moshkovskii* may cause inflammation in the gut and create a niche that helps in the survival and proliferation of other enteropathogenic bacteria/viroses".
In line 594 "The observed multiple infections with many helminths and protozoan parasites could be explained by their same mode of transmission and poor hygiene areas.
In line 596 " Co-infection of *E. moshkovskii* and *E. histolytica* also suggests the absence of competitive exclusion among them. We also did not document any invasive disease in the diarrheal patients infected with *E. moshkovskii".*

The authors should providing proper context for your research and avoiding introducing new information and also put references

In line *598 "*More interestingly, the *E. moshkovskii* and *E. histolytica* co-infected subgroup also did not show any invasive disease indicating the parallel evolution of these two species. Therefore, the mystery is to be revealed under what conditions *E. moshkovskii* cells turn pathogenic, as many diarrhoeal incidences were observed that were solely infected with this species. This capability may result from particular parasite genetics and/or genotypes in *E. moshkovskii* isolates.

Our answer:

Your comments are greatly appreciated. We have removed all of the sentences that seem to be overinterpreted. We would be grateful to receive any further input or comments you may have regarding this change. Your feedback is valuable to us.
