# Supplementary material for: Prevalence and molecular characterization of Entamoeba moshkovskii in diarrheal patients from Eastern India
Source: PLoS Negl Trop Dis. 2023 May 11;17(5):e0011287. doi: 10.1371/journal.pntd.0011287 (PMC10218735; doi:10.1371/journal.pntd.0011287)
Supplement: Attachment — Submitted filename: Response to the authors_PLOS NEGLECTED_PNTD-D-22-01293R1.docx [file pntd.0011287.s004.docx]

Manuscript Number: PNTD-D-22-01293R1

Dear authors

The content of the manuscript has value for publication at a local scale, but must be modified in order to make this paper suitable for publication in PLOS Neglected Tropical Diseases.

I summarize my comment below based on the general context

The authors must correct all types grammatical error in manuscript. For instance, in line 563, replace “dirrhoeal” by diarrhoeal

As mentioned by authors, in line 362 , the authors argued that “ Interestingly, 100% (n/N=17/17, 95% CI 0.784-363 1.00) of trophozoites were found to be non-hematophagous, indicating that *E. moshkovskii* may cause a non invasive form of intestinal amoebic infection”

This information is redundant because of globally it is observed that 90% of E. histolytica infections are asymptomatic and only in case of invasive amebiasis is observed hematophagous trophozoites on stool microscopy.

In line 319 , item Data collection of other Enteropathogen infections:

The authors mention: " Entamoeba histolytica, Cryptosporidium, and Giardia were identified by PCR method as described elsewhere. Helminth parasites were detected by light microscopy after wet mount. Other enteric pathogens like Vibrio cholera O1/O139, Salmonella spp., Campylobacter jejuni, Rotavirus, astrovirus and adenovirus co-infection information with entamoeba were obtained from the institutional database of ICMR-National Institute of Cholera and Enteric Diseases, Kolkata, India. Detailed information is available elsewhere [41]".

This is reference is about a hospital-based laboratory surveillance study that it was conducted among the patients admitted between November 2007 and October 2008 to the Infectious Diseases (ID) Hospital (Population = 1103) with diarrhoeal complaints.

Could the author explain about it. Where is detailed information about of present paper data (hospital-based systemic surveillance data conducted from March 2017 to February 2020)?

In line 374, diarrheagenic organisms were screened in the *E. moshkovskii-positive* diarrheal cases. There are no mention about methodology for virus and bacteria detections. What were methods employed?

In line 392 item GIS Mapping: The authors mentioned

“GIS mapping of the patients admitted to the I.D. hospital and B C Roy Children Hospital pointed out that the Beliaghata, Entally, Kashipur-Belgachia, Maniktala, Gopalpur-Rajarhat, Kolkata-Port, Jorasanko, Baruipurpurba, Maheshtala, Metiabruz, Bhangar areas of Kolkata and the adjacent regions were very highly infected (>3.5%) with *Entamoeba moshkovskii*. Ballygunge, Chowragee, Dumdum Uttar, Dumdum, Bidhannagar, Bhabanipur and Kashba had high prevalence rate (3%-3.5%)*.* Behala Paschim, Behala Purba, Jadavpur were moderately (2.5%-2.99%) infected, while the lowest prevalence (< 2.49%) was reported in Baranagar, Rajarhat New Town and Shyampukur areas [Figure 3]. Those areas that constituted less than 1% of the total sample size have been excluded from the mapping”.

I suggest put prevalence rate in figure GIS Mapping and the authors must reduce this paragraph in order to avoid replicate information. Images help readers visualize the information.

In line 408, the authors mentioned “the rest of the 32 sequences represented genetic variants of *E. moshkovskii* not described earlier”

How many nucleotide sequences (genetic variants of E. moshkovskii ) are currently avaliable by GenBank?

intraspecific polymorphism exist but this information should be analysed based on robust data

In line 587 the authors mentioned that “SNPs in the 18S rRNA gene could have a significant impact on the biology of the amoeba”

Could the authors explain about it ? what is significant consequence ?

Could the authors place this setence within the context of previous studies

In discussion section, the authors should compare and contrast to previous studies. And also, although interpretation is the primary goal of the Discussion section, authors must be careful not to overinterpret their data, or stray too far from scientific evidence.

The author should providing proper context for your research and avoiding introducing new information

For instances, in line 592 “*E. moshkovskii* infection may alter susceptibility to infection with other enteric pathogens and modulate the effects of co-infecting gut pathogens”.

In line 593 “*E. moshkovskii* may cause inflammation in the gut and create a niche that helps in the survival and proliferation of other enteropathogenic bacteria/viroses”.

In line 594 “The observed multiple infections with many helminths and protozoan parasites could be explained by their same mode of transmission and poor hygiene areas.

In line 596 “ Co-infection of *E. moshkovskii* and *E. histolytica* also suggests the absence of competitive exclusion among them. We also did not document any invasive disease in the diarrheal patients infected with *E. moshkovskii”.*

The authors should providing proper context for your research and avoiding introducing new information and also put references

In line *598 “*More interestingly, the *E. moshkovskii* and *E. histolytica* co-infected subgroup also did not show any invasive disease indicating the parallel evolution of these two species. Therefore, the mystery is to be revealed under what conditions *E. moshkovskii* cells turn pathogenic, as many diarrhoeal incidences were observed that were solely infected with this species. This capability may result from particular parasite genetics and/or genotypes in *E. moshkovskii* isolates.
