# Supplementary material for: Prevalence and molecular characterization of Entamoeba moshkovskii in diarrheal patients from Eastern India
Source: PLoS Negl Trop Dis. 2023 May 11;17(5):e0011287. doi: 10.1371/journal.pntd.0011287 (PMC10218735; doi:10.1371/journal.pntd.0011287)
Supplement: Attachment — Submitted filename: Response to the authors_PLOS NEGLECTED_PNTD-D-22-01293R2.docx [file pntd.0011287.s006.docx]

Manuscript Number: PNTD-D-22-01293R2

Dear authors

The content of the manuscript has value for publication at a local scale, but must be modified in order to make this paper suitable for publication in PLOS Neglected Tropical Diseases.

My recommendation: Minor revision

Figures are an important part of research. They visually convey your findings to support how the authors verbally describe your research.

Please do not hesitate to contact me if you have any questions.
